# The Effect of Vitamin D Supplementation on Glycemic Control and Cardiovascular Risk Factors in Type 2 Diabetes: An Updated Systematic Review and Meta-Analysis of Clinical Trials

**DOI:** 10.1155/2024/9960656

**Published:** 2024-09-10

**Authors:** Maryam Afraie, Pourya Bahrami, Parisa Kohnepoushi, Sorour Khateri, Lobat Majidi, Lotfollah Saed, Kamran Zamani, Hedyeh Mohammadi Baharm, Yousef Moradi, Farhad Moradpour

**Affiliations:** ^1^ Student Research Committee Kurdistan University of Medical Sciences, Sanandaj, Kurdistan, Iran; ^2^ Department of Physical Medicine and Rehabilitation Hamedan University of Medical Sciences, Hamedan, Iran; ^3^ Department of Internal Medicine School of Medicine Kurdistan University of Medical Sciences, Sanandaj, Iran; ^4^ Social Determinants of Health Research Center Research Institute for Health Development University of Medical Sciences, Sanandaj, Kurdistan, Iran

**Keywords:** meta-analysis, systematic review, Type 2 diabetes, vitamin D supplementation

## Abstract

**Background and Aims:** The purpose of this meta-analysis was to investigate the effect of vitamin D supplementation on hemoglobin A1C (HbA1C), fasting blood sugar (FBS), low-density lipoprotein (LDL), high-density lipoprotein (HDL), systolic blood pressure (SBP), and the total vitamin D level in patients with Type 2 diabetes (T2DM).

**Methods:** A systematic search was conducted in databases such as PubMed (Medline), Scopus, Embase, Web of Science, Cochrane Library, and ClinicalTrials.gov using relevant keywords from January 1990 to January 2024. After screening and extracting data, a qualitative evaluation of articles was performed using the Cochrane risk-of-bias tool for randomized trials (RoB 2).

**Results:** The findings revealed that vitamin D supplementation significantly decreased the mean HbA1C (SMD: −0.15; 95% CI: −0.29, −0.20; *I*_square_: 79.76%; *p* value < 0.001) and mean FBS (SMD: −0.28; 95% CI: −0.40, −0.15; *I*_square_: 70.13%; *p* value < 0.001), lowered SBP (SMD: −0.06; 95% CI: −0.16, −0.05; *I*_square_: 39.63%; *p* value = 0.23), and reduced LDL (SMD: −0.11; 95% CI: −0.28, −0.05; *I*_square_: 73.66%; *p* value < 0.001). Furthermore, vitamin D supplementation increased the average HDL (SMD: 0.13; 95% CI: 0.04, 0.29; *I*_square_: 79.33%; *p* value < 0.001) and vitamin D levels (SMD: 1.78; 95% CI: 1.53, 2.04; *I*_square_: 91.92%; *p* value < 0.001) in patients with T2DM. Subgroup analyses showed that weight gain, BMI, and duration of the disease could reduce the effect of vitamin D supplementation on diabetes control in affected patients.

**Conclusion:** The results also indicated that taking vitamin D supplements in the amount of 50,000 IU had a significant effect on reducing the indicators related to diabetes control. Based on the combined evidence, the findings of this meta-analysis suggest that vitamin D supplementation can significantly improve glycemic control and reduce the risk of complications associated with T2DM, especially cardiovascular diseases (CVDs).

## 1. Introduction

Type 2 diabetes (T2DM) is a prevalent and progressive metabolic disorder among adults worldwide, carrying high mortality and morbidity rates. In 2021, 529 million people worldwide lived with diabetes, and the age-standardized prevalence was 6.1%, with a confidence interval ranging from 5.8% to 6.5%. The highest age-standardized prevalence was observed in North Africa and the Eastern Mediterranean. In 2021, the leading risk factor for diabetes incidence was high body mass index (BMI), indicating that this risk factor contributed significantly to the attributable risk. Projections suggest that by 2050, 89 out of 204 countries worldwide will have an age-standardized prevalence exceeding 10% [1]. T2DM ranked as the seventh leading cause of disability (in terms of DALYs) and the ninth leading cause of death globally. These alarming statistics highlight the need for effective interventions to manage and prevent the complications associated with T2DM [2–4]. Approximately one-third of the US population is estimated to be at risk of developing T2DM or being in a prediabetic state. Each year, 10% of individuals with prediabetes progress to T2DM, underscoring the need for preventive measures and interventions for early detection and management of the disease [4, 5]. The mechanism of T2DM is characterized by insufficient secretion of insulin from pancreatic *β*-cells and an inadequate response of insulin-sensitive tissues to insulin. This can arise due to a combination of factors, such as genetic background, obesity, aging, and physical inactivity [6–8]. Classic clinical symptoms of diabetes include polyuria, polyphagia, and polydipsia, which, if undiagnosed and untreated, can lead to various complications such as retinopathy, neuropathy, nephropathy, peripheral vascular disease, and cerebrocardiovascular diseases (CVDs). Diagnostic tests used to screen for diabetes in individuals without classic hyperglycemia symptoms include fasting blood sugar (FBS), hemoglobin A1c (HbA1C), and 2-h plasma glucose concentration. According to the latest update from the American Diabetes Association, a diagnosis of diabetes requires two abnormal screening tests in cases where classic clinical symptoms of diabetes are absent [9]. Vitamin D is a prohormone of the secosteroid type, which is mostly produced in the body after sunlight exposure to the skin. After being produced in the skin, it needs to be hydroxylated twice to become the active form, or 1.25 (OH)2D, respectively, in the liver and kidneys [10, 11]. Reasons such as reducing the duration of exposure to sunlight, improper diet, accelerated losses of vitamin D, and impaired activation of vitamin D led to hypovitaminosis D and, as a result, symptoms such as muscle pain, arthralgia, fatigue and weakness, osteoporosis, and rickets [12–15]. In recent years, several studies have been conducted on the effect of vitamin D supplementation on blood sugar control and the prevention of complications due to its high level (blood sugar) in patients with T2DM. In a clinical trial conducted by López et al. in 2016 on 140 patients with T2DM, patients who received vitamin D along with a drug regimen had lower HbA1C levels after 3 months than patients who received a placebo [16]. In another clinical trial conducted by Khan et al. on 90 patients with T2DM, after 2 months, the patients who received vitamin D had a lower HbA1C level [17]. Other similar studies have been conducted that have reported the positive effect of vitamin D supplementation on the reduction of HbA1C [18–20]. Clinical trials have also been conducted on the effect of vitamin D on FBS control. For example, in two clinical trials conducted by Omidian et al. and Upreti et al. on 48 and 60 people, respectively, after 3 months of follow-up, the group that received vitamin D had a lower FBS level [21]. Although several studies have reported a positive effect of vitamin D supplementation on blood sugar control in patients with T2DM, other studies have reported conflicting findings [22, 23]. In a clinical trial conducted by El Hajj et al. in 2018 on 88 patients with T2DM, the group receiving vitamin D supplementation did not show a significant difference in the level of HbA1C compared to the group receiving placebo after the follow-up period [24]. In several studies, it is possible that vitamin D, in addition to its anti-inflammatory effect as well as its effect on intracellular enzyme pathways through blood sugar control, can reduce complications such as retinopathy and nephropathy in T2DM patients [25, 26]. Given the high incidence, prevalence, morbidity, and mortality of patients with T2DM, along with conflicting reports on the effect of vitamin D supplementation on blood sugar levels and the potential benefits of vitamin D in preventing complications associated with T2DM, this meta-analysis aims to determine the effect of vitamin D supplementation on the average indices of HbA1C, FBS, low-density lipoprotein (LDL), high-density lipoprotein (HDL), systolic blood pressure (SBP), and the overall vitamin D level in patients with T2DM. The findings of this meta-analysis have the potential to inform and update treatment and care guidelines related to T2DM.

## 2. Methods

This systematic review and meta-analysis was conducted in accordance with the preferred reporting items for systematic reviews and meta-analyses (PRISMA) guidelines [27]. Additionally, the protocol for this systematic review and meta-analysis was registered in the international prospective register of systematic reviews (PROSPERO) to ensure transparency and reduce the risk of bias (CRD42023401300).

The study's keywords were selected based on the research question and objectives and then combined using AND with OR operators to formulate the search strategy and syntax for each database. The main search terms included vitamin D and its synonyms (calcitriol, cholecalciferol, vitamin D, vitamin D2, and vitamin D3) and T2DM and its synonyms (diabetes, T2D, and hyperglycemia) (Table S1). Thesauruses, Emtree, and Mesh databases were used to identify additional synonyms. The databases searched included PubMed (Medline), Scopus, Embase, Web of Science, Cochrane Library, and ClinicalTrials.gov, covering the period from January 1990 to January 2024. To ensure that no relevant studies were missed, a manual search was conducted to identify additional studies by checking the references and sources cited in the selected studies. After completing the search, the retrieved studies were imported into EndNote Version 8 software and screened based on the title, abstract, and full text using the inclusion and exclusion criteria. This process was conducted by two independent reviewers (H.M.B. and M.A.), and any discrepancies were resolved through discussion or consultation with a third reviewer. To enhance the reliability of this process, the authors independently double-checked the references manually, ensuring further confidence in the results. The inclusion criteria for this systematic review and meta-analysis were based on the PICOT structure. Studies were considered if they involved patients with T2DM as the population, and the main intervention was vitamin D supplementation, with a comparison group receiving a placebo or other routine drugs. The desired outcomes included average HbA1C, FBS, LDL, HDL, SBP, and vitamin D levels in the body after taking vitamin D supplements in patients. The mean differences in serum vitamin D levels were chosen as one of the primary endpoints of the study based on the indications from the selected trials in the search results, verifying the inclusion of oral vitamin D supplements and identifying serum vitamin D levels as a key focus of the research. Only interventional studies, specifically randomized clinical trials, were considered for inclusion. The exclusion criteria included articles with repeated citations, review articles, cross-sectional studies, case-control or cohort studies, books, conference articles, and clinical trials with different primary and intervention outcomes. Additionally, clinical trials that did not consider vitamin D supplementation as an intervention, did not involve patients with T2DM, or did not report a standardized mean difference (SMD) as the measurement index were excluded from the study. The screening process was conducted by two independent reviewers (M.A. and F.M.), and any discrepancies were resolved through discussion or consultation with a third reviewer (Y.M.).

After the screening, the quality assessment of the selected studies was done using Version 2 of the Cochrane risk-of-bias tool for randomized trials (RoB 2) [28]. The RoB 2 tool evaluates the risk of bias in five domains, including the randomization process, deviations from intended interventions, missing outcome data, measurement of the outcome, and selection of the reported result. For each domain, the study was rated as low, high, or unclear risk of bias. By using the RoB 2 tool, the authors were able to assess the quality of the selected studies and ensure that the study findings were reliable and accurate.

Finally, extracting information by examining items related to studies (name of authors, year of publication, type of study, sample size, and country of study), items related to the target population (age, BMI, and duration of diabetes), the items related to the intervention (the type of intervention and how to do it) of the comparison group, and, finally, the desired outcomes (average HbA1C, FBS, LDL, HDL, SBP, and vitamin D level of the body) were performed. The three stages of selecting studies, qualitative assessment, and data extraction were done independently by two authors (M.A./H.M.B. and F.M.), and any discrepancies were resolved through discussion or consultation with a third reviewer (Y.M.).

STATA Version 17 software was used to perform the meta-analysis. The desired index for the analysis was the SMD. To calculate this index, before and after the intervention, the average and standard deviation (SD) for each outcome in each group were extracted from the selected studies, and their differences were calculated. Then, using STATA software Version 17, this index was calculated by considering the fixed effect model. Egger's test and funnel plot were used to evaluate publication bias. Also, the *I*_square_ index and the *Q* Cochrane test were used to evaluate heterogeneity. Subgroup analyses were performed based on age, BMI, duration of diabetes, different doses of supplements, continent, and length of follow-up of patients (based on weeks). Metaregression analysis was also performed on the basis of patients' BMI and age to assess the effect of these two variables on the relationship between vitamin D supplementation and diabetes control. The significance level in this meta-analysis was considered below 0.05. The grading of recommendations assessment, development, and evaluation (GRADE) approach was used to assess the overall quality of evidence for each outcome listed in the summary of findings table. The GRADEpro GDT online software was used to conduct the GRADE approach and generate a summary of findings tables (Table S2).

## 3. Results

**Figure 1:**
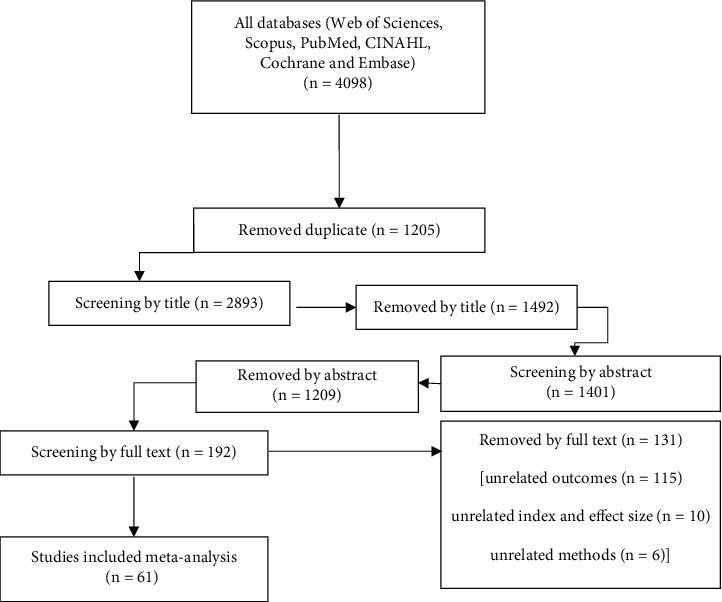
A flow diagram demonstrating the study selection process.

At the outset of this study, a total of 4098 articles were identified through a comprehensive search process. Following a rigorous screening process based on the title and abstract, 1205 and 1492 articles were excluded, respectively. Subsequently, 192 articles were subjected to full-text screening, and after careful evaluation, 131 articles were excluded due to unrelated outcome (115 articles), unrelated effect size (10 articles), and unrelated methodology (six articles). Finally, 61 clinical trials that met the inclusion criteria were selected for the meta-analysis (Figure 1).  Table 1 presents a comprehensive overview of the characteristics of the included randomized controlled trials. All relevant details, such as study design, sample size, intervention, and outcome measures, have been carefully documented to facilitate a thorough understanding of the studies included in this meta-analysis.

### 3.1. HbA1C

The first outcome of this meta-analysis was determining the effect of vitamin D supplementation on average HbA1C. To achieve this goal, the average HbA1C before and after the intervention was reported in 45 studies out of all clinical trial studies selected in this meta-analysis. From these studies, without considering subgroup analyses, 61 effect sizes (SMD) were extracted and considered for meta-analysis. After combining these results, the SMD was equal to −0.15 (SMD: −0.15; 95% CI: −0.29, −0.20; *I*_square_: 79.76%; *p* value < 0.001) (Table 2).

Subgroup analyses were performed based on continent, age, BMI, duration of diabetes, different doses of vitamin D, and duration of follow-up in selected clinical trial studies. The results showed that the effect of vitamin D supplementation on reducing the average HbA1C in Asian patients was more than in European patients (SMD: −0.32; 95% CI: −0.53, −0.11; *I*_square_: 84.18%; *p* value < 0.001). But in individuals with diabetes living in America, this impression was the opposite, in the form of an increase in the average HbA1C (SMD: 0.30; 95% CI: −0.19, 0.79; *I*_square_: 88.08%; *p* value < 0.001) (Table 2). Subgroup analysis based on the age of individuals with diabetes and BMI showed that the effect of vitamin D supplementation in individuals with diabetes less than 60 years old and a BMI of less than 30 is significantly greater in reducing the average HbA1C. Also, if the duration of diabetes in patients is more than 10 years, vitamin D reduces HbA1C more (SMD: −0.49; 95% CI: −1.19, 0.21; *I*_square_: 88.49%; *p* value < 0.001), but it is not statistically significant (Table 2). Concerning the duration of follow-up in selected clinical trial studies after the intervention, subgroup analyses showed that in the case of 8-week follow-up after the intervention, the effect of vitamin D on reducing HbA1C is greater than other follow-ups such as 16, 36, or 48 weeks (SMD: −0.38; 95% CI: −0.88, 0.12; *I*_square_: 90.30%; *p* value < 0.001). After the 8th week, the follow-up up to the 12th week also showed the effect of vitamin D on the reduction of HbA1C, but after this week, it showed different effects of vitamin D on the control of HbA1C (Table 2). Subgroup analysis based on different doses of vitamin D reveals that receiving a dose less than 50,000 IU has shown a greater effect on reducing HbA1C (SMD: −0.22; 95% CI: −0.46, 0.02; *I*_square_: 86.44%; *p* value < 0.001) (Table 2).

**Figure 2:**
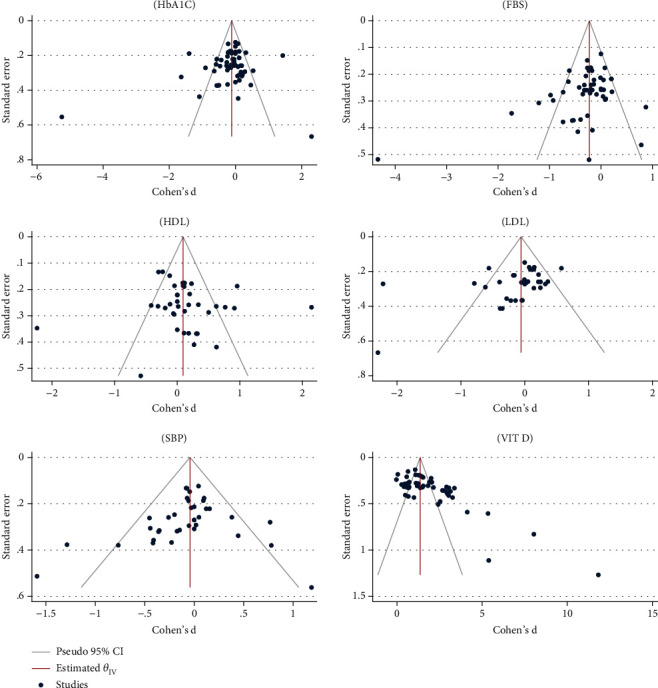
The results of publication bias based on funnel plot about effect of vitamin D supplemental on the control of diabetes.

Metaregression analysis was performed to determine the effect of age and BMI variables on the effect of vitamin D supplementation on average HbA1C, and the results showed that the effect of vitamin D on average HbA1C increases with the age of patients (*β*: 0.20; SE: 0.01; 95% CI: −0.09, 0.05; *p*: 0.185), and with the increase of BMI, this effect decreases (*β*: −0.49; SE: 0.04; 95% CI: −0.13, 0.03; *p*: 0.271). Egger's test analyses to check publication bias showed that this bias did not occur in the overall analysis of determining the effect of vitamin D on average HbA1C (*β*: −1.48; SE: 0.80; *p*: 0.06) (Figure 2).

### 3.2. Fasting Blood Glucose

The second objective was to assess the impact of vitamin D supplementation on mean FBS. In total, 40 studies with 50 reported effect sizes were included in the meta-analysis. Upon combining these findings, the SMD was calculated as −0.28 (SMD: −0.28; 95% CI: −0.40, −0.15; *I*_square_: 70.13%; *p* value < 0.001). This indicates a statistically significant reduction in FBS levels among individuals with diabetes (Table 2).

Subgroup analyses showed that the effect of vitamin D supplementation on the reduction of mean FBS in the Asian community (SMD: −0.30; 95% CI: −0.47, −0.12; *I*_square_: 77.56%; *p* value < 0.001) and patients with BMI less than 30 (SMD: −0.24; 95% CI: −0.38, −0.09; *I*_square_: 54.51%; *p* value < 0.001) is more (Table 2). Based on the duration of diabetes in patients, most of the included studies included patients with a duration of disease of less than 10 years. After combining these results, vitamin D supplementation reduced the average FBS to 0.14. Based on different doses of vitamin D, the dose of 50,000 IU (SMD: −0.44; 95% CI: −0.69, −0.20; *I*_square_: 78.84%; *p* value < 0.001) and follow-up for 24 weeks after the intervention (SMD: −0.38; 95% CI: −0.64, −0.12; *I*_square_: 81.61%; *p* value < 0.001) have shown a better effect of vitamin D on reducing the average FBS (Table 2).

Metaregression analysis was performed to determine the effect of age and BMI variables on the effect of vitamin D supplementation on average FBS, and the results showed that with age increasing (*β*: 0.02; SE: 0.00; 95% CI: −0.01, 0.03; *p*: 0.672) and BMI increasing (*β*: 0.01; SE: 0.00; 95% CI: −0.02, 0.04; *p*: 0.577) in patients, the effect of vitamin D on average FBS increases. Egger's test analyses for checking publication bias showed that this bias occurred in the overall analysis of determining the effect of vitamin D on average FBS (*β*: −2.50; SE: 0.81; *p* value ≤ 0.001) (Figure 2).

### 3.3. SBP

The next outcome in this meta-analysis was mean SBP. The reason for not reporting diastolic blood pressure (DBP) was not reporting its average in selected studies. Based on the analysis, vitamin D supplementation has reduced the mean SBP by 0.06 (SMD: −0.06; 95% CI: −0.16, −0.05; *I*_square_: 39.63%; *p* value = 0.23) (Table 3).

According to the continent, this effect in individuals with diabetes living in Europe (SMD: −0.06; 95% CI: −0.21, 0.08; *I*_square_: 48.58%; *p* value = 0.12) is more than in Asians (SMD: −0.05; 95% CI: −0.20, 0.09; *I*_square_: 27.65%; *p* value = 0.15). Also, this effect if the age of individuals with diabetes is less than 60 years (SMD: −0.08; 95% CI: −0.21, −0.05; *I*_square_: 25.51%; *p* value = 0.16) and the BMI is more than 30 (SMD: −0.07; 95% CI: −0.25, −0.11; *I*_square_: 43.76%; *p* value = 0.05) is different and has a greater reducing effect on the average SBP. Based on different doses of vitamin D, a dose of 50,000–100,000 IU (SMD: −0.08; 95% CI: −0.22, −0.06; *I*_square_: 31.56%; *p* value = 0.12) and follow-up for 8 weeks after the intervention (SMD: 0.45; 95% CI: −0.94, −0.04; *I*_square_: 64.03%; *p* value = 0.04) have shown a better effect of vitamin D on reducing the average SBP (Table 3).

Metaregression analysis was performed to determine the effect of age and BMI variables on the effect of vitamin D supplementation on average SBP, and the results showed that with age increasing (*β*: 0.20; SE: 0.05; 95% CI: −0.06, 0.42; *p*: 0.705) and BMI increasing (*β*: 0.23; SE: 0.10; 95% CI: −0.01, 0.56; *p*: 0.816) in patients, the effect of vitamin D on average HbA1C increases. Egger's test analyses to check publication bias showed that this bias did not occur in the overall analysis of determining the effect of vitamin D on mean SBP (*β*: −0.87; SE: 0.61; *p*: 0.16) (Figure 2).

### 3.4. HDL

The effect of vitamin D on average HDL in patients with diabetes was investigated and reported in 30 selected clinical trial studies. From these studies, 38 effect sizes (SMD) were extracted and combined. The results of the meta-analysis showed that, in general, vitamin D consumption increases the average HDL by 0.13 (SMD: 0.13; 95% CI: 0.04, 0.29; *I*_square_: 79.33%; *p* value < 0.001) (Table 4).

Based on the continent, patients' age, and BMI, the results of subgroup analyses showed that this effect in patients with diabetes living in America (SMD: 0.59; 95% CI: 0.12, 1.30; *I*_square_: 87.39%; *p* value < 0.001), age over 60 years (SMD: 0.37; 95% CI: 0.12, 0.61; *I*_square_: 78.09%; *p* value < 0.001), and BMI less than 30 years (SMD: 0.19; 95% CI: 0.02, 0.41; *I*_square_: 77.81%; *p* value < 0.001) is more and more significant. Based on different doses of vitamin D, the results showed that the consumption of vitamin D of fewer than 50,000 IU has a more significant effect on increasing the average HDL (SMD: 0.15; 95% CI: 0.09, 0.39; *I*_square_: 81.19%;*p* value < 0.001). Also, the follow-up for 16 weeks after the intervention (SMD: 0.53; 95% CI: 0.33, 1.19; *I*_square_: 87.65%; *p* value < 0.001) has shown better the effect of vitamin D on reducing the average HDL (Table 4).

Metaregression analysis to determine the effect of age variables and BMI on the vitamin D supplementation effect on mean HDL showed that with age increasing (*β*: 0.08; SE: 0.02; 95% CI: 0.01, 0.13; *p*: <0.001), the effect of vitamin D on average HDL increases, while this effect with increasing BMI (*β*: −0.06; SE: 0.03; 95% CI: −0.12, −0.01; *p*: 0.04) decreases. Egger's test analyses for checking publication bias showed that this bias did not occur in the overall analysis of determining the effect of vitamin D on mean SBP (*β*: −0.22; SE: 1.08; *p*: 0.84) (Figure 2).

### 3.5. LDL

Twenty-seven clinical trial studies with 34 effect sizes (SMD) were included in the analysis related to determining the effect of vitamin D consumption on mean LDL. The results of the meta-analysis showed that the consumption of vitamin D reduces the mean LDL to 0.11 (SMD: −0.11; 95% CI: −0.28, −0.05; *I*_square_: 73.66%; *p* value < 0.001) (Table 4).

Subgroup analyses based on age, BMI, duration of diabetes, different doses of vitamin D, and duration of follow-up are shown in Table 2. Based on the different doses of vitamin D and the duration of follow-up, the results showed that a dose of fewer than 50,000 IU (SMD: −0.23; 95% CI: −0.52, −0.06; *I*_square_: 82.19%; *p* value < 0.001) and a follow-up period of 24 weeks after taking vitamin D (SMD: −0.26; 95% CI: −0.64, −0.13; *I*_square_: 86.61%; *p* value < 0.001) show the effect of this vitamin on reducing the average LDL better (Table 4).

The metaregression analysis for determining the effect of age variables and BMI on the effect of vitamin D supplementation on mean LDL showed that with age increasing (*β*: 0.03; SE: 0.01; 95% CI: −0.01, 0.09; *p*: 0.581) in patients, the effect of vitamin D on the mean LDL increases, while this effect decreases with increasing BMI (*β*: −0.01; SE: 0.00; 95% CI: −0.02, 0.00; *p*: 0.167). Egger's test analyses for checking publication bias showed that this bias occurred in the overall analysis of determining the effect of vitamin D on mean SBP (*β*: −2.68; SE: 1.01; *p*: <0.001) (Figure 2).

### 3.6. Vitamin D

In another part of the present meta-analysis results, the average level of vitamin D in the body of individuals with diabetes was measured and analyzed before and after taking vitamin D supplements. In this analysis, the SMD of the average level of vitamin D in the body in two groups receiving vitamin D supplements and placebo was calculated and combined. Finally, the result of the meta-analysis showed that the average level of vitamin D in the body increased by 1.78 IU (SMD: 1.78; 95% CI: 1.53, 2.04; *I*_square_: 91.92%; *p* value < 0.001) (Table 5).

Based on subgroup analyses, the average vitamin D level after taking vitamin D supplements in patients with diabetes, living in Asia, less than 60 years old, BMI less than 30, and length of diabetes of less than 10 years is higher. Also, the dose of 50,000 IU of vitamin D supplement had a greater effect on increasing the vitamin D level in the body than other doses (Table 5). Metaregression analysis to determine the effect of age and BMI variables on the effect of vitamin D supplementation on average vitamin D showed that with age increasing (*β*: −0.04; SE: 0.02; 95% CI: −0.09, −0.01; *p*: <0.001) and BMI increasing (*β*: −0.05; SE: 0.04; 95% CI: −0.22, −0.01; *p*: 0.02), this effect decreases. Egger's test analyses to check publication bias showed that this bias occurred in the overall analysis of determining the effect of vitamin D on mean SBP (*β*: 8.12; SE: 0.76; *p*: <0.001) (Figure 2).

### 3.7. Risk of Bias

**Figure 3:**
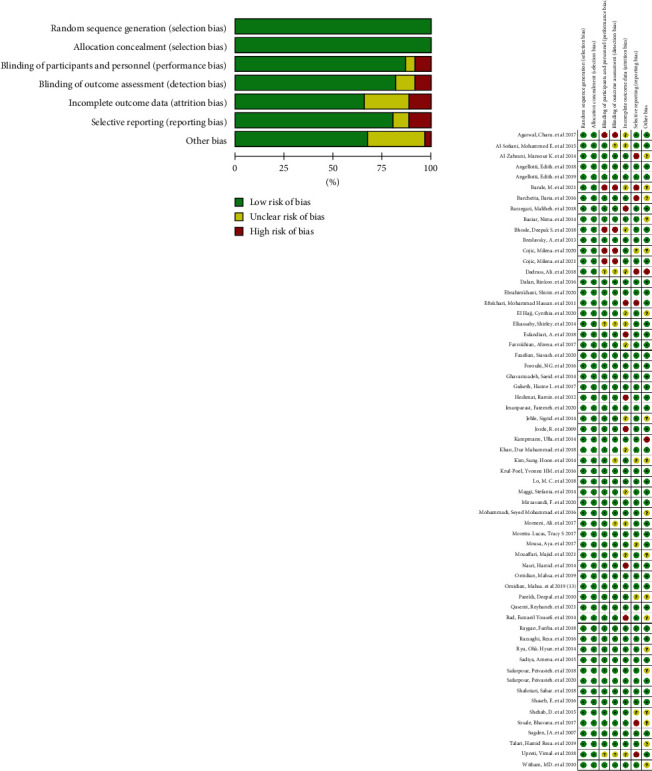
Risk of bias graph and summary (review authors' judgments about each risk of bias item presented as percentages across all included studies).

Figure 3 provides a visual representation of the results of the RoB 2 assessment conducted in this study. The figure presents a comprehensive overview of the risk of bias for each included study, including the domains of bias assessed and the corresponding judgments. Overall, the results of the RoB 2 assessment highlight the methodological quality of the included studies and provide a framework for the interpretation of the findings presented in this meta-analysis (Figure 3). Also, the results of the quality assessment based on GRADE indicate that the overall quality of results for various outcomes in this meta-analysis is at a level of moderate to high. This signifies the adequate quality of the calculated and reported results in this meta-analysis (Table S2).

## 4. Discussion

The main goal of this meta-analysis was to determine the effect of vitamin D supplementation on patients with T2DM. The results showed that vitamin D supplementation has a significant effect on diabetes control and the reduction of blood pressure and blood lipid indicators in patients with T2DM. The average indices of HbA1C and FBS have decreased by 0.15 and 0.28, respectively, in patients with T2DM who take vitamin D supplements. The most common complications of individuals with diabetes are cardiovascular problems, and research results have shown the importance of vitamin D deficiency in the prevalence of CVD, and they have confirmed that this vitamin is a therapeutic or preventive factor in T2DM. Vitamin D is one of those fat-soluble vitamins that can be stored in the body, and its excessive consumption can cause complications such as calcium deposition in the kidneys, lungs, heart, and ears; bone pain; loss of appetite and nausea; increased urine volume; constipation; kidney disorders; and even poisoning [29]. Therefore, despite all the benefits of vitamin D, excessive use of this vitamin can have an adverse effect on human health. Vitamin D deficiency is associated with increased BMI, higher blood pressure, high triglycerides, and insulin resistance, all of which predispose a person to T2DM. The important point is that receiving optimal amounts of vitamin D affects the prevention or control of diabetes, and its excessive consumption is associated with serious complications [30–33]. Vitamin D receptors are present in almost every cell of the body, which indicates the important role of this vitamin in the body's chemical processes and the reduction of HbA1C and FBS indices [29, 34]. HbA1C is caused by the continuous, slow glycosylation of nonenzymatic hemoglobin caused by hyperglycemia. A prospective study of diabetes in England showed that HbA1C is a gold standard for evaluating blood sugar control in T2DM, and in addition, reducing its value by 1% can lead to a 14% reduction in the occurrence of previous vascular diseases [35, 36]. George, Pearson, and Witham conducted a meta-analysis to evaluate the effect of vitamin D on blood glucose control and insulin resistance, the results of which showed that vitamin D leads to a small improvement in fasting plasma glucose (FBG) and insulin resistance, but no beneficial effect on HbA1C was observed [37]. The results of the present meta-analysis showed that vitamin D supplementation prevents the increase of plasma HbA1C, which indicates that the effect of vitamin D is to reduce or delay the occurrence and development of diabetes complications. These conflicting results may be due to the increase in studies and the more detailed and updated analysis of the present meta-analysis. In 2019, researchers conducted a meta-analysis of intervention trials and discovered that vitamin D supplementation reduced FBG but had variable effects on insulin secretion and HbA1C [38]. Another meta-analysis discovered that vitamin D supplementation dramatically reduced HbA1C levels, potentially delaying or preventing diabetic consequences. However, the same study discovered that the positive effects of vitamin D may be confined to vitamin D–deficient people [39]. A 2023 systematic review and meta-analysis suggest that vitamin D may improve FPG, HbA1C, and homeostasis model assessment-insulin resistance (HOMA-IR) in people with T2DM. In addition, a 2021 meta-analysis showed that vitamin D supplementation improved fasting insulin levels [40, 41]. A variety of factors, including study population characteristics, vitamin D levels utilized, and evidence quality, could explain the contradictory findings on vitamin D's impact on blood glucose management and insulin resistance in T2DM and prediabetes.

In the last decade, vitamin D deficiency in T2DM and serum level of 25-hydroxyvitamin D have been considered predictors of long-term complications of diabetes such as CVD. This vitamin plays a significant role in regulating the function of pancreatic beta cells (insulin-secreting cells in the pancreas). In T2DM, the beta cells try to produce more insulin because there is resistance to insulin in other tissues of the body, and this hormone cannot have the necessary function. Among the most important factors that cause insulin resistance are overweight, lack of physical activity, high abdominal fat, and genetic background [42–45]. The results of the current meta-analysis indicate that as BMI decreases, the impact of vitamin D supplementation on reducing HbA1C and FBS indices becomes more pronounced. This finding supports the hypothesis of a negative correlation between weight gain and insulin resistance, suggesting that vitamin D supplementation or maintaining sufficient levels of vitamin D in individuals with T2DM may mitigate the development of insulin resistance. However, it is worth noting that further investigation is warranted to explore whether the effectiveness of vitamin D supplementation in reducing HbA1C and FBS indices diminishes with weight gain. Additional studies with adequate sample sizes are needed to address this hypothesis comprehensively.

Vitamin D deficiency increases the level of LDL and ultimately increases the risk of CVD in patients with T2DM. Therefore, it can be said that vitamin D deficiency can increase the complications of diabetes, especially CVD, and ultimately increase death and the direct and indirect health costs of the disease [46–49]. The results of the present meta-analysis showed that vitamin D supplementation can significantly reduce SBP and LDL levels in patients with T2DM. Therefore, it can be claimed that its consumption can be effective in preventing serious complications of T2DM such as CVD.

Several meta-analyses have been conducted to assess the effect of vitamin D in different forms, such as supplements or prescription medicines, and at different doses, on glycemic control. However, it is important to emphasize that most of these trials have focused on people with prediabetes, and few trials have been conducted in more diverse populations. These meta-analyses provide information about the potential effects of vitamin D on glycemic control and the prevention of T2DM. However, they highlight the need for more high-quality research to address the limitations and inconsistencies in the existing evidence. In the subgroup analyses, the meta-analysis results showed that the effect of vitamin D supplementation is affected by increasing age, BMI, and the duration of diabetes, and there may be a significant effect like its effect in patients with T2DM. With an age of less than 60 years, a BMI of less than 30, and the duration of diabetes not less than 10 years. The reason for this can be the increase in the body's resistance to vitamin D supplementation or the interference of judgment in old age due to the use of other drugs to prevent important and chronic diseases of old age. Another point is that in subgroup analyses, the amount of heterogeneity has decreased significantly in some subgroups.

In the present meta-analysis, the level of vitamin D in the body increased significantly after the use of vitamin D supplements in patients with T2DM. Subgroup analyses related to this analysis also showed that this average was higher in patients living in Asia, less than 60 years old, BMI of less than 30, and duration of diabetes of fewer than 10 years. Also, the dose of 50,000 IU of vitamin D supplement had a greater effect on increasing the level of vitamin D in the body than other doses. One of the strengths of this meta-analysis compared to previous meta-analyses was the calculation of the average level of vitamin D in the body after taking vitamin D supplements in patients with T2DM, which can confirm the increase in the level of vitamin D in the body after using the supplement and then reduce complications related to T2DM. Vitamin D is actually a preventative measure and chemically is considered a steroid hormone. The fact that insulin is also a hormone convinces some experts that there is a relationship between insulin and vitamin D, and it also seems that many people with low vitamin D levels also develop general immunodeficiency, which can increase their incidence of diabetes and other diseases. Other strengths of this study include the large number of studies included in the analysis and the accuracy of the reported estimates. It also performed subgroup analyses based on important variables such as vitamin D dose, duration of diabetes, and duration of follow-up after the intervention. Among the weaknesses of the present meta-analysis, we can point out the lack of analysis of other indicators related to diabetes, such as DBP, ethnicity, and HOMA-IR, which was due to the lack of full reporting of these indicators in selected studies. It is recommended that in future intervention studies regarding the impact of vitamin use on blood glucose control, race be considered as the primary variable for conducting subgroup analyses.

## 5. Conclusion

The findings of this meta-analysis suggest that vitamin D supplementation can significantly decrease indicators related to T2DM and, subsequently, reduce the risk of complications, particularly CVDs. Therefore, the adoption of a care program aimed at T2DM prevention and management could include supplemental vitamin D consumption, dietary intake, or other methods such as regular sun exposure. Furthermore, vitamin D could be considered as a potential addition to treatment guidelines for patients with T2DM. The combined evidence presented in this study highlights the significant potential of vitamin D as a preventive and therapeutic intervention for T2DM and its associated complications.

## Figures and Tables

**Table 1:** The characteristics of included randomized control trials in this meta-analysis.

**Authors (years)**	**Years**	**Country**	**Sample size intervention**	**Sample size placebo**	**Mean age**	**BMI**	**Disease duration**	**Follow-up duration**	**Intervention**	**Study population**
Barale et al. (2021) [50]	2021	Italy	14	16	71.5	29.8	NR	12 weeks; 24 weeks; 36 weeks; 48 weeks	5000 IU/day	T2DM
Cojic et al. (2021) [51]	2021	Serbia	65	65	60	30	4	12 weeks; 24 weeks	50,000 IU/week	T2DM
Mozaffari et al. (2021) [52]	2021	Iran	40	40	NR	NR	NR	12 weeks	50,000 IU/week	T2DM
Safarpour et al. (2020) [53]	2020	Iran	42	43	48.3	31.37	NR	8 weeks	50,000 IU/week	T2DM
Angellotti et al. (2018) [54]	2018	United States	61	59	60.2	30.9	NR	16 weeks; 24 weeks; 36 weeks; 48 weeks	4000 IU/day	Patients with well-controlled Type 2 diabetes (metformin or lifestyle)
Khan et al. (2018) [17]	2018	Pakistan	70	70	54.8	NR	>10	12 weeks	50,000 IU/week	Patients with Type 2 diabetes with vitamin D deficiency
Safarpour et al. (2018) [55]	2018	Iran	42	43	50.36	30.43	5.5	8 weeks	50,000 IU/week	T2DM
Sosale et al. (2017) [56]	2017	India	29	31	53	25.9	7	24 weeks	60,000 IU/week	Type 2 diabetes and dyslipidemia, had A1c below 9%, and low vitamin D levels (<30 ng/mL)
Upreti et al. (2018) [23]	2018	India	30	30	48.3	24.56	NR	24 weeks	60,000 IU/week	Patients with coexisting Type 2 diabetes mellitus and hypovitaminosis D
Angellotti et al. (2019) [57]	2019	United States	66	61	60.1	30.7	NR	24 weeks; 48 weeks	4000 IU/day	Patients with well-controlled Type 2 diabetes (metformin or lifestyle)
Esfandiari et al. (2018) [58]	2018	Iran	25	25	39.7	NR	NR	8 weeks	50,000 IU/week	T2DM
Omidian et al. (2019) [59]	2019	Iran	32	34	49.7	27.3	NR	12 weeks	4000 IU/day	T2DM
Omidian et al. (2019) [21]	2019	Iran	23	23	51.3	26.8	5.8	12 weeks	4000 IU/day	T2DM
Talari et al. (2019) [60]	2019	Iran	30	31	67.3	29.2		24 weeks	50,000 IU/week	T2DM
Cojic et al. (2020) [61]	2020	Montenegro	50	68	60	29.34		12 weeks; 24 weeks	50,000 IU/week	T2DM+metformin
El Hajj et al. (2020) [24]	2020	Lebanon	45	43	66.9	22.6	8.7	24 weeks	30,000 IU/week	T2DM
Mirzavandi et al. (2020) [62]	2020	Iran	25	25	45.56	31	7	8 weeks	200,000 IU/once	T2DM
Jorde and Figenschau (2009) [63]	2009	Norway	16	16	56.25	32.1		24 weeks	40,000 IU/week	T2DM
Eftekhari et al. (2011) [64]	2011	Iran	35	35	53.8	28.3		12 weeks; 10 weeks	5000 IU/day	T2DM
Heshmat et al. (2012) [65]	2012	Iran	21	21	56.2	27.7	6.7	12 weeks	300,000 IU/once	T2DM+metformin+glibenclamide
Nasri et al. (2014) [66]	2014	Iran	30	30	55.1			12 weeks	50,000 IU/week	T2DM
Jehle et al. (2014) [20]	2014	Switzerland	29	26	66.9	28.9	12.7	12 weeks; 24,weeks	300,000 IU/once	T2DM+metformin+sulfonylureas+pioglitazone+GLP-1 receptor signaling+insulin
Park et al. (2014) [67]	2014	Korea	11	13	73.27	24.08		12 weeks	1200 IU/day	T2DM
Ryu et al. (2014) [68]	2014	Korea	64	65	54.8	25	5.7	24 weeks	1000 IU/day	T2DM+meglitinide+metformin+alpha-glucosidase inhibitor+sulfonylurea+pioglitazone
Rad et al. (2014) [69]	2014	Iran	28	30	50.03	27.94	5.89	8 weeks	4000 IU/day	T2DM+metformin+glibenclamide
Al-Zahrani et al. (2014) [70]	2014	Saudi	91	92	56.9	31.3	NR	12 weeks	45,000 IU/week	T2DM+metformin Insulin
Mohammadi et al. (2016) [71]	2016	Iran	28	25	38.5	28.8	NR	12 weeks	50,000 IU/week	T2DM
Forouhi et al. (2016) [72]	2016	United Kingdom	114	112	53.5	28.9	NR	16 weeks	20,000 IU/week	T2DM
Lo, Abushamat, and Mramba (2018) [73]	2018	United States	14	16	NR	NR	NR	24 weeks	50,000 IU/week	T2DM
Breslavsky et al. (2013) [74]	2013	Israel	24	23	66.8	27.9	NR	48 weeks	1000 IU/day	T2DM+metformin+sulfonilure+repaglinid+DDP-4 inhibitors
Dadrass et al. (2018) [75]	2018	Iran	12	12	53.7	27.7	NR	12 weeks	5000 IU/day	T2DM
Bhosle and Mubeen (2018) [18]	2018	India	60	60	NR	NR	NR	12 weeks; 24 weeks	60,000 IU/week	T2DM
Sadiya et al. (2015) [76]	2015	UAE	43	39	49.8	37.9	9.5	24 weeks; 12 weeks	3000 IU/day	T2DM
Sugden et al. (2007) [77]	2007	United Kingdom	17	17	64.9	31.7		8 weeks	100,000 IU/once	T2DM+metformin+sulfonylureas+insulin+thiazolidinedione
Momeni et al. (2017) [78]	2017	Iran	30	30	62.4			8 weeks	50,000 IU/week	T2DM
Baziar et al. (2014) [79]	2014	Iran	41	40	50.34	27.33	5.44	8 weeks	50,000 IU/week	T2DM
Razzaghi et al. (2016) [80]	2016	Iran	30	30	59.6	26		12 weeks	50,000 IU/week	T2DM+metformin+insulin
Dalan et al. (2016) [81]	2016	Singapore	31	30	52.2	27.3		16 weeks	4000 IU/day	T2DM+metformin+sulfonilure+DDP-4 inhibitors
Witham et al. (2010) [82]	2010	United Kingdom	19	22	65.3	31.1		8 weeks; 16 weeks	100,000 IU/once	T2DM
									200,000 IU/once	
Shaseb et al. (2016) [83]	2016	Iran	48	47	54.9	27.2	3.9	8 weeks	300,000 IU/once	T2DM
Ghavamzadeh, Mobasseri, and Mahdavi (2014) [84]	2014	Iran	26	25	52.26	28.9		14 weeks	400 IU/day	T2DM+metformin+insulin
Krul-Poel et al. (2016) [29]	2016	Netherlands	129	132	67	28.7	6	24 weeks	50,000 IU/week	T2DM+metformin+sulfonylurea
Barzegari et al. (2018) [85]	2018	Iran	25	25	39.7	21.1		8 weeks	50,000 IU/week	T2DM
Kampmann et al. (2014) [86]	2014	Denmark	7	8	61.6	33.8	5.6	12 weeks	5600 IU/day	T2DM+insulin
Farrokhian et al. (2017) [87]	2017	Iran	30	30	61.7	30.2		24 weeks	50,000 IU/week	T2DM
Shahriari, Eftekhari, and Jeddi (2018) [88]	2018	Iran	28	29	57.1	27.6	6.9	8 weeks	50,000 IU/week	T2DM
Parekh et al. (2010) [89]	2010	India	14	13	42.36	23.54	4.8	4 weeks	300,000 IU/once	T2DM+metformin+glibenclamide
Gulseth et al. (2017) [90]	2017	Norway	33	29	55.5	31.8	11.9	24 weeks	400,000 IU/once	T2DM+metformin, sulfonylureas, pioglitazone, exenatide, and dipeptidyl peptidase 4 inhibitors
Imanparast et al. (2020) [91]	2020	Iran	23	23	53.63	28.29		16 weeks	50,000 IU/week	T2DM+glibenclamide or repaglinide+metformin or only metformin
Agarwal, Marwah, and Kulshrestha (2017) [92]	2017	India	30	30	57.13			12 weeks	60,000 IU/week	T2DM
Al-Sofiani et al. (2015) [22]	2015	Saudi Arabia	10	10	54.8		9.3	12 weeks	5000 IU/day	T2DM
Barchetta et al. (2016) [93]	2016	Italy	26	29	57.4	29.3	5.9	24 weeks	2000 IU/day	T2DM
Elkassaby et al. (2014) [94]	2014	Australia	26	24				12 weeks; 24 weeks	6000 IU/day	T2DM+metformin
Ebrahimkhani, Ghavamzadeh, and Mehdizadeh (2020) [95]	2020	Iran	17	19	50.53	30.2	6.5	12 weeks	50,000 IU/week	T2DM
Fazelian et al. (2020) [96]	2020	Iran	26	25	48.5	30.21		16 weeks	50,000 IU/week	T2DM
Maggi et al. (2014) [97]	2014	Italy	12	13	69	29		12 weeks; 24 weeks	300,000 IU/once	T2DM
Moreira-Lucas et al. (2017) [98]	2017	Canada	35	36	49.1	30.1		24 weeks	28,000 IU/week	T2DM
Mousa et al. (2017) [99]	2017	Australia	28	26	30.5	30.5		16 weeks	100,000 IU/once	T2DM
Nasri et al. (2014) [66]	2014	Iran	30	30	55	29.3		12 weeks	50,000 IU/week	T2DM
Qasemi et al. (2021) [100]	2021	Iran	23	21	55.13		9.83	12 weeks	2000 IU/day	T2DM+metformin+glibenclamide+glitazone
Raygan et al. (2018) [101]	2018	Iran	30	30	71.5	29		12 weeks	50,000 IU/week	T2DM
Shehab et al. (2015) [102]	2015	Kuwait	57	55	61.8			8 weeks	50,000 IU/week	T2DM

**Table 2:** The overall effect of vitamin D supplement on the HbA1C and FBS in patients with diabetes based on continent, age, BMI, diabetes duration, dose of intervention, and follow-up duration.

**Variables**		**Category**	**No. study**	**Pooled MSD (95% CI)**	**Heterogeneity assessment between studies**			**Heterogeneity assessment between subgroup**		**Publication bias assessments**		
					**I** ^2^	**p** ** value**	**Q**	**Q**	**p** ** value**	**β**	**SE**	**p** ** value**
HbA1C	Overall SMD		45 (61)	−0.15 (−0.29, −0.2)	79.76%	≤0.001	296.37	—	—	−1.48	0.806	0.065
	Continent	Europe	12 (22)	−0.08 (−0.17, 0.02)	5.45%	0.39	22.21	7.08	0.03			
		Asia	30 (33)	−0.32 (−0.53, −0.11)	84.18%	≤0.001	102.27					
		America	3 (6)	0.30 (−0.19, 0.79)	88.08%	≤0.001	41.94					
	Age	<60	28 (30)	−0.35 (−0.56, −0.13)	84.56%	≤0.001	187.79	8.44	≤0.001			
		>60	13 (25)	0.05 (−0.11, 0.22)	66.83%	≤0.001	72.36					
	BMI	<30	22 (28)	−0.32 (−0.52, −0.11)	80.92%	≤0.001	141.48	6.63	0.01			
		>30	12 (20)	0.06 (−0.15, 0.26)	75.76%	≤0.001	78.38					
	Disease duration	<10	14 (16)	−0.18 (−0.49, −0.13)	86.44%	≤0.001	110.64	0.62	0.43			
		>10	3 (4)	−0.49 (−1.19, 0.21)	88.49%	≤0.001	26.07					
	Intervention	<50,000	20 (29)	−0.22 (−0.46, 0.02)	86.38%	≤0.001	205.55	0.78	0.68			
		50,000–100,000	22 (25)	−0.15 (−0.31, −0.02)	72.25%	≤0.001	86.50					
		>100,000	5 (7)	−0.08 (−0.28, 0.12)	0.00	0.84	2.76					
	Follow-up duration	8 weeks	10 (11)	−0.38 (−0.88, 0.12)	90.30%	≤0.001	103.08	30.53	≤0.001			
		12 weeks	19 (19)	−0.29 (−0.51, −0.06)	75.25%	≤0.001	72.73					
		14 weeks	1 (1)	−1.63 (−2.28, −1.01)	—	—	—					
		16 weeks	5 (7)	−0.02 (−0.17, 0.12)	0.00%	0.76	3.38					
		24 weeks	18 (18)	−0.03 (−0.13, 0.07)	0.00%	0.68	13.87					
		36 weeks	2 (2)	0.62 (−1.01, 2.26)	93.69%	≤0.001	15.85					
		48 weeks	3 (3)	−0.07 (−0.36, −0.21)	3.63%	0.35	2.08					

FBS	Overall SMD		40 (50)	−0.28 (−0.40, −0.15)	70.13%	≤0.001	164.07	—	—	−2.50	0.812	0.002
	Continent	Europe	8 (13)	−0.19 (−0.31, −0.07)	0.00%	0.83	7.42	1.12	0.57			
		Asia	31 (36)	−0.30 (−0.47, −0.12)	77.56%	≤0.001	155.97					
		America	1 (1)	−0.14 (−0.61, 0.33)	—	—	—					
	Age	<60	26 (29)	−0.19 (−0.34, −0.04)	63.01%	≤0.001	75.70	0.00	1.00			
		>60	12 (17)	−0.19 (−0.30, −0.08)	0.00%	0.60	13.96					
	BMI	<30	22 (28)	−0.24 (−0.38, −0.09)	54.51%	≤0.001	59.35	0.96	0.33			
		>30	12 (14)	−0.14 (−0.26, −0.02)	2.92%	0.42	13.39					
	Disease duration	<10	16 (18)	−0.14 (−0.34, 0.06)	71.02%	≤0.001	58.65	0.26	0.61			
		>10	1 (1)	0.00 (−0.50, 0.50)	—	—	—					
	Intervention	<50,000	17 (24)	−0.44 (−0.69, −0.2)	78.84%	≤0.001	108.70	5.23	0.07			
		50,000–100,000	19 (22)	−0.21 (−0.34, −0.08)	45.65%	0.01	38.64					
		>100,000	4 (4)	0.12 (−0.34, 0.57)	67.71%	0.03	9.29					
	Follow-up duration	8 weeks	9 (9)	−0.25 (−0.58, 0.08)	76.01%	≤0.001	33.35	1.34	0.97			
		12 weeks	18 (19)	−0.25 (−0.43, −0.06)	60.43%	≤0.001	45.49					
		10 weeks	1 (1)	−0.19 (−0.66, 0.28)	—	—	—					
		16 weeks	2 (2)	−0.15 (−0.57, 0.28)	14.88%	0.28	1.17					
		24 weeks	16 (16)	−0.38 (−0.64, −0.12)	81.61%	≤0.001	81.58					
		36 weeks	1 (1)	−0.40 (−1.12, 0.33)	—	—	—					
		48 weeks	2 (2)	−0.19 (−0.83, 0.45)	47.51%	0.17	1.91					

**Table 3:** The overall effect of vitamin D supplement on the SBP in patients with diabetes based on continent, age, BMI, diabetes duration, dose of intervention, and follow-up duration.

**Variables**		**Category**	**No. study**	**Pooled MSD (95% CI)**	**Heterogeneity assessment between studies**			**Heterogeneity assessment between subgroup**		**Publication bias assessments**		
					**I** ^2^	**p** ** value**	**Q**	**Q**	**p** ** value**	**β**	**SE**	**p** ** value**
SBP	Overall SMD		26 (37)	−0.06 (−0.16, 0.05)	39.63%	0.23	59.63			−0.87	0.617	0.160
	Continent	Europe	11 (21)	−0.06 (−0.21, 0.08)	48.58%	0.12	38.89	0.01	0.94			
		Asia	15 (16)	−0.05 (−0.20, 0.09)	27.65%	0.15	20.73					
	Age	<60	15 (17)	−0.08 (−0.21, −0.05)	25.51%	0.16	21.44	0.14	0.71			
		>60	11 (20)	−0.04 (−0.20, 0.12)	49.47%	0.01	37.60					
	BMI	<30	15 (21)	−0.02 (−0.13, 0.00)	21.30%	0.19	25.41	0.21	0.64			
		>30	9 (14)	−0.07 (−0.25, −0.11)	43.76%	0.05	23.12					
	Disease duration	<10	11 (13)	0.01 (−0.15, 0.18)	37.59%	0.08	19.23	0.87	0.35			
		>10	1 (2)	0.38 (−0.37, 1.13)	74.29%	0.05	3.89					
	Intervention	<50,000	13 (18)	−0.06 (−0.22, 0.10)	45.11%	0.02	30.97	0.45	0.80			
		50,000–100,000	11 (14)	−0.08 (−0.22, −0.06)	31.56%	0.12	18.99					
		>100,000	3 (5)	0.06 (−0.32, 0.45)	54.13	0.07	8.72					
	Follow-up duration	8 weeks	3 (4)	−0.45 (−0.94, −0.04)	64.03%	0.04	8.34	8.19	0.15			
		12 weeks	14 (14)	−0.05 (−0.23, 0.13)	43.29%	0.04	22.94					
		16 weeks	4 (6)	−0.04 (−0.20, 0.11)	0.00%	0.68	3.10					
		24 weeks	10 (10)	−0.00 (−0.16, 0.16)	23.75%	0.22	11.80					
		36 weeks	1 (1)	0.78 (0.04, 1.52)	—	—	—					
		48 weeks	2 (2)	−0.34 (−1.11, −0.43)	63%	0.10	2.70					

**Table 4:** The overall effect of vitamin D supplement on the HDL and LDL in patients with diabetes based on continent, age, BMI, diabetes duration, dose of intervention, and follow-up duration.

**Variables**		**Category**	**No. study**	**Pooled MSD (95% CI)**	**Heterogeneity assessment between studies**			**Heterogeneity assessment between subgroup**		**Publication bias assessments**		
					**I** ^2^	**p** ** value**	**Q**	**Q**	**p** ** value**	**β**	**SE**	**p** ** value**
HDL	Overall SMD		30 (38)	0.13 (0.04–0.29)	79.33%	≤0.001	179.01	—	—	−0.22	1.084	0.840
	Continent	Europe	7 (13)	0.07 (0.01–0.13)	0.00%	0.51	11.23	5.21	0.07			
		Asia	22 (23)	0.16 (0.11–0.42)	84.00%	≤0.001	137.51					
		America	1 (2)	0.59 (0.12–1.30)	87.39%	≤0.001	7.93					
	Age	<60	18 (20)	0.10 (0.04–0.17)	71.68%	≤0.001	67.09	7.86	0.01			
		>60	12 (18)	0.37 (0.12–0.61)	78.09%	≤0.001	77.58					
	BMI	<30	19 (24)	0.19 (0.02–0.41)	77.81%	≤0.001	103.64	0.94	0.33			
		>30	10 (13)	0.01 (−0.30 to 0.31)	84.06%	≤0.001	75.29					
	Disease duration	<10	10 (12)	0.12 (−0.22 to 0.46)	83.85%	≤0.001	68.12	—	—			
	Intervention	<50,000	15 (21)	0.15 (0.09–0.39)	81.19%	≤0.001	106.32	0.10	0.95			
		50,000–100,000	14 (16)	0.10 (0.03–0.35)	79.28%	≤0.001	72.40					
		>100,000	1 (1)	0.12 (0.06–0.44)	—	—	—					
	Follow-up duration	8 weeks	6 (6)	0.10 (0.03–0.30)	0.00%	0.46	4.65	8.54	0.13			
		12 weeks	11 (11)	0.11 (0.05–0.28)	19.17%	0.26	12.37					
		16 weeks	5 (5)	0.53 (0.33–1.19)	87.65%	≤0.001	32.40					
		24 weeks	12 (12)	0.31 (0.04–0.67)	84.86%	≤0.001	72.67					
		36 weeks	1 (1)	0.32 (0.40–1.04)	—	—	—					
		48 weeks	3 (3)	0.43 (0.25–1.11)	78.71%	0.01	9.39					

LDL	Overall SMD		27 (34)	−0.11 (−0.28 to −0.05)	73.66%	≤0.001	125.15	—	—	−2.68	1.015	0.008
	Continent	Europe	20 (21)	0.00 (−0.20 to 0.20)	37.04%	0.10	15.88	1.08	0.58			
		Asia	6 (11)	−0.16 (−0.38 to 0.07)	76.85%	≤0.001	86.41					
		America	1 (2)	0.01 (−1.11 to 1.12)	94.92%	≤0.001	19.68					
	Age	<60	16 (17)	−0.07 (−0.21 to 0.07)	28.81%	0.13	22.48	0.38	0.54			
		>60	11 (17)	−0.17 (−0.47 to 0.13)	84.43%	≤0.001	102.79					
	BMI	<30	17 (21)	−0.16 (−0.41 to 0.09)	77.04%	≤0.001	87.11	0.37	0.54			
		>30	9 (12)	−0.05 (−0.28 to 0.17)	68.88%	≤0.001	35.35					
	Disease duration	<10	9 (11)	−0.32 (−0.72 to 0.09)	87.60%	≤0.001	80.62	—	—			
	Intervention	<50,000	14 (19)	−0.23 (−0.52 to −0.06)	82.19%	≤0.001	101.09	2.10	0.15			
		50,000–100,000	13 (15)	0.01 (−0.13 to 0.15)	30.75%	0.12	20.22					
	Follow-up duration	8 weeks	5 (5)	−0.02 (−0.27 to 0.24)	32.52%	0.20	5.93	9.48	0.09			
		12 weeks	11 (11)	−0.17 (−0.40, −0.07)	56.46%	0.01	22.97					
		16 weeks	3 (3)	0.18 (−0.13, 0.49)	0.00%	0.62	0.95					
		24 weeks	11 (11)	−0.26 (−0.64, −0.13)	86.61%	≤0.001	74.67					
		36 weeks	1 (1)	−0.04 (−0.76, −0.67)	—	—	—					
		48 weeks	3 (3)	0.37 (0.03–0.71)	23.83%	0.27	2.63					

**Table 5:** The overall effect of vitamin D supplement on the vitamin D level in patients with diabetes based on continent, age, BMI, diabetes duration, dose of intervention, and follow-up duration.

**Variables**		**Category**	**No. study**	**Pooled MSD (95% CI)**	**Heterogeneity assessment between studies**			**Heterogeneity assessment between subgroup**		**Publication bias assessments**		
					**I** ^2^	**p** ** value**	**Q**	**Q**	**p** ** value**	**β**	**SE**	**p** ** value**
VIT D	Overall SMD		48 (59)	1.78 (1.53–2.04)	91.92%	≤0.001	718.6	—	—	8.12	0.760	≤0.001
	Continent	Europe	9 (16)	1.10 (0.85–1.36)	71.05%	≤0.001	51.81	18.53	≤0.001			
		Asia	39 (43)	2.05 (1.70–2.40)	93.48%	≤0.001	644.13					
	Age	<60	33 (35)	1.86 (1.51–2.22)	91.71%	≤0.001	410.18	3.73	0.05			
		>60	13 (19)	1.38 (1.05–1.72)	87.23%	≤0.001	140.96					
	BMI	<30	25 (30)	1.83 (1.45–2.21)	92.35%	≤0.001	379.13	0.54	0.46			
		>30	13 (18)	1.62 (1.22–2.02)	88.85%	≤0.001	152.52					
	Disease duration	<10	18 (20)	2.00 (1.57–2.42)	91.70%	≤0.001	229.01	8.71	≤0.001			
		>10	1 (2)	0.92 (0.35–1.50)	52.24%	0.15	2.09					
	Intervention	<50,000	15 (18)	2.36 (1.73–2.99)	94.62%	≤0.001	315.72	29.76	≤0.001			
		50,000–100,000	27 (31)	1.78 (1.46–2.09)	91.09%	≤0.001	336.73					
		>100,000	7 (10)	0.86 (0.59–1.13)	45.26%	0.06	16.44					
	Follow-up duration	4 weeks	1 (1)	2.42 (1.42–3.41)	—	—	—	41.82	≤0.001			
		8 weeks	11 (12)	1.48 (1.08–1.88)	84.02%	≤0.001	68.82					
		12 weeks	22 (23)	2.15 (1.64–2.67)	94.18%	≤0.001	377.92					
		14 weeks	1 (1)	3.03 (2.23–3.84)	—	—	—					
		16 weeks	6 (6)	1.26 (0.53–1.99)	87.56%	≤0.001	40.19					
		24 weeks	15 (15)	1.85 (1.36–2.33)	92.73%	≤0.001	192.62					
		48 weeks	1 (1)	0.27 (−0.30 to 0.85)	—	—	—					

## Data Availability

The datasets used during and/or analyzed during the current study are available from the corresponding authors on reasonable request.

## Supporting Information

Supporting Information Additional supporting information can be found online in the Supporting Information section. Supporting Information details the search queries used to identify relevant studies, incorporating various keywords and MeSH terms across databases. The GRADE assessment indicates moderate to high confidence in the evidence, suggesting that vitamin D supplementation may significantly improve glycemic control and cardiovascular risk factors in T2DM patients.
