# Supplementary material for: The Effect of Vitamin D Supplementation on Glycemic Control and Cardiovascular Risk Factors in Type 2 Diabetes: An Updated Systematic Review and Meta-Analysis of Clinical Trials
Source: J Diabetes Res. 2024 Sep 10;2024:9960656. doi: 10.1155/2024/9960656 (PMC11407890; doi:10.1155/2024/9960656)
Supplement: Supporting Information — Additional supporting information can be found online in the Supporting Information section. Supporting Information details the search queries used to identify relevant studies, incorporating various keywords and MeSH terms across databases. The GRADE assessment indicates moderate to high confidence in the evidence, suggesting that vitamin D supplementation may significantly improve glycemic control and cardiovascular risk factors in T2DM patients. [file 9960656.f1.docx]

Supplementary file, Table 1: Search syntaxes and query.

| Databases | Search Query | Results |
| --- | --- | --- |
| PubMed | (((("vitamin d"[MeSH Terms] OR "vitamin d"[All Fields] OR "ergocalciferols"[MeSH Terms] OR "ergocalciferols"[All Fields]) AND ("supplemental"[All Fields] OR "supplementating"[All Fields] OR "supplementation"[All Fields] OR "supplementation s"[All Fields] OR "supplementations"[All Fields] OR "supplemention"[All Fields])) OR ("vitamin d"[MeSH Terms] OR "vitamin d"[All Fields] OR "ergocalciferols"[MeSH Terms] OR "ergocalciferols"[All Fields]) OR ("ergocalciferols"[MeSH Terms] OR "ergocalciferols"[All Fields] OR ("vitamin"[All Fields] AND "d2"[All Fields]) OR "vitamin d2"[All Fields]) OR ("cholecalciferol"[MeSH Terms] OR "cholecalciferol"[All Fields] OR ("vitamin"[All Fields] AND "d3"[All Fields]) OR "vitamin d3"[All Fields]) OR ("cholecalciferol"[MeSH Terms] OR "cholecalciferol"[All Fields] OR "cholecalciferols"[All Fields] OR "colecalciferol"[All Fields]) OR ("ergocalciferols"[MeSH Terms] OR "ergocalciferols"[All Fields] OR "ergocalciferol"[All Fields]) OR ("alfacalcidol"[Supplementary Concept] OR "alfacalcidol"[All Fields] OR "alphacalcidol"[All Fields]) OR ("alfacalcidol"[Supplementary Concept] OR "alfacalcidol"[All Fields]) OR ("paricalcitol"[Supplementary Concept] OR "paricalcitol"[All Fields]) OR ("1 alpha hydroxyergocalciferol"[Supplementary Concept] OR "1 alpha hydroxyergocalciferol"[All Fields] OR "doxercalciferol"[All Fields]) OR ("calcitriol"[MeSH Terms] OR "calcitriol"[All Fields] OR "calcitriols"[All Fields]) OR ("25 hydroxyvitamin d"[Supplementary Concept] OR "25 hydroxyvitamin d"[All Fields] OR "25 hydroxyvitamin d"[All Fields] OR "calcifediol"[MeSH Terms] OR "calcifediol"[All Fields]) OR ("vitamin d"[MeSH Terms] OR "vitamin d"[All Fields] OR "ergocalciferols"[MeSH Terms] OR "ergocalciferols"[All Fields])) AND ("diabete"[All Fields] OR "diabetes mellitus"[MeSH Terms] OR ("diabetes"[All Fields] AND "mellitus"[All Fields]) OR "diabetes mellitus"[All Fields] OR "diabetes"[All Fields] OR "diabetes insipidus"[MeSH Terms] OR ("diabetes"[All Fields] AND "insipidus"[All Fields]) OR "diabetes insipidus"[All Fields] OR "diabetic"[All Fields] OR "diabetics"[All Fields] OR "diabets"[All Fields] OR ("diabetes mellitus"[MeSH Terms] OR ("diabetes"[All Fields] AND "mellitus"[All Fields]) OR "diabetes mellitus"[All Fields]) OR "T2DM"[All Fields] OR ("hyperglycaemia"[All Fields] OR "hyperglycemia"[MeSH Terms] OR "hyperglycemia"[All Fields] OR "hyperglycaemias"[All Fields] OR "hyperglycemias"[All Fields] OR "hyperglycemia s"[All Fields]) OR ("hyperglycaemia"[All Fields] OR "hyperglycemia"[MeSH Terms] OR "hyperglycemia"[All Fields] OR "hyperglycaemias"[All Fields] OR "hyperglycemias"[All Fields] OR "hyperglycemia s"[All Fields]) OR ("glucose"[MeSH Terms] OR "glucose"[All Fields] OR "glucoses"[All Fields] OR "glucose s"[All Fields]) OR ("glycated hemoglobin"[MeSH Terms] OR ("glycated"[All Fields] AND "hemoglobin"[All Fields]) OR "glycated hemoglobin"[All Fields] OR "hba1c"[All Fields] OR "hba1cs"[All Fields]) OR ("glycated hemoglobin"[MeSH Terms] OR ("glycated"[All Fields] AND "hemoglobin"[All Fields]) OR "glycated hemoglobin"[All Fields]) OR ("insulin resistance"[MeSH Terms] OR ("insulin"[All Fields] AND "resistance"[All Fields]) OR "insulin resistance"[All Fields]) OR ("insulin resistance"[MeSH Terms] OR ("insulin"[All Fields] AND "resistance"[All Fields]) OR "insulin resistance"[All Fields] OR ("insulin"[All Fields] AND "sensitivity"[All Fields]) OR "insulin sensitivity"[All Fields]) OR "HOMA"[All Fields] OR (("glucose"[MeSH Terms] OR "glucose"[All Fields] OR "glucoses"[All Fields] OR "glucose s"[All Fields]) AND ("homoeostasis"[All Fields] OR "homeostasis"[MeSH Terms] OR "homeostasis"[All Fields])) OR ("insulin secretion"[MeSH Terms] OR ("insulin"[All Fields] AND "secretion"[All Fields]) OR "insulin secretion"[All Fields]) OR ("insulin"[MeSH Terms] OR "insulin"[All Fields] OR "insulin s"[All Fields] OR "insuline"[All Fields] OR "insulinic"[All Fields] OR "insulinization"[All Fields] OR "insulinized"[All Fields] OR "insulins"[MeSH Terms] OR "insulins"[All Fields]) OR (("insulin secreting cells"[MeSH Terms] OR ("insulin secreting"[All Fields] AND "cells"[All Fields]) OR "insulin secreting cells"[All Fields] OR ("beta"[All Fields] AND "cell"[All Fields]) OR "beta cell"[All Fields]) AND ("functional"[All Fields] OR "functional s"[All Fields] OR "functionalities"[All Fields] OR "functionality"[All Fields] OR "functionalization"[All Fields] OR "functionalizations"[All Fields] OR "functionalize"[All Fields] OR "functionalized"[All Fields] OR "functionalizes"[All Fields] OR "functionalizing"[All Fields] OR "functionally"[All Fields] OR "functionals"[All Fields] OR "functioned"[All Fields] OR "functioning"[All Fields] OR "functionings"[All Fields] OR "functions"[All Fields] OR "physiology"[MeSH Subheading] OR "physiology"[All Fields] OR "function"[All Fields] OR "physiology"[MeSH Terms])) OR ("glycemic control"[MeSH Terms] OR ("glycemic"[All Fields] AND "control"[All Fields]) OR "glycemic control"[All Fields]) OR (("glucose"[MeSH Terms] OR "glucose"[All Fields] OR "glucoses"[All Fields] OR "glucose s"[All Fields]) AND ("immune tolerance"[MeSH Terms] OR ("immune"[All Fields] AND "tolerance"[All Fields]) OR "immune tolerance"[All Fields] OR "tolerance"[All Fields] OR "drug tolerance"[MeSH Terms] OR ("drug"[All Fields] AND "tolerance"[All Fields]) OR "drug tolerance"[All Fields] OR "tolerabilities"[All Fields] OR "tolerability"[All Fields] OR "tolerable"[All Fields] OR "tolerableness"[All Fields] OR "tolerably"[All Fields] OR "tolerances"[All Fields] OR "tolerant"[All Fields] OR "tolerants"[All Fields] OR "tolerate"[All Fields] OR "tolerated"[All Fields] OR "tolerates"[All Fields] OR "tolerating"[All Fields] OR "toleration"[All Fields] OR "tolerator"[All Fields] OR "tolerators"[All Fields] OR "tolerence"[All Fields])) OR (("glucose"[MeSH Terms] OR "glucose"[All Fields] OR "glucoses"[All Fields] OR "glucose s"[All Fields]) AND ("metabolic"[All Fields] OR "metabolical"[All Fields] OR "metabolically"[All Fields] OR "metabolics"[All Fields] OR "metabolism"[MeSH Terms] OR "metabolism"[All Fields] OR "metabolisms"[All Fields] OR "metabolism"[MeSH Subheading] OR "metabolities"[All Fields] OR "metabolization"[All Fields] OR "metabolize"[All Fields] OR "metabolized"[All Fields] OR "metabolizer"[All Fields] OR "metabolizers"[All Fields] OR "metabolizes"[All Fields] OR "metabolizing"[All Fields])) OR (("fasted"[All Fields] OR "fasting"[MeSH Terms] OR "fasting"[All Fields] OR "fastings"[All Fields] OR "fasts"[All Fields]) AND ("blood glucose"[MeSH Terms] OR ("blood"[All Fields] AND "glucose"[All Fields]) OR "blood glucose"[All Fields])) OR ("front biosci"[Journal] OR "fbs"[All Fields]) OR ("diabetes mellitus"[MeSH Terms] OR ("diabetes"[All Fields] AND "mellitus"[All Fields]) OR "diabetes mellitus"[All Fields]) OR ("glycated hemoglobin"[MeSH Terms] OR ("glycated"[All Fields] AND "hemoglobin"[All Fields]) OR "glycated hemoglobin"[All Fields] OR "glycated hemoglobin a"[All Fields]) OR ("insulin resistance"[MeSH Terms] OR ("insulin"[All Fields] AND "resistance"[All Fields]) OR "insulin resistance"[All Fields]))) AND (clinicaltrial[Filter] OR randomizedcontrolledtrial[Filter]) | 1825 |
| Web of Sciences | 1# ====="vitamin D supplementation" (Title) or "vitamin D" (Title) or "vitamin D2" (Title) or "vitamin D3" (Title) or cholecalciferol (Title) or ergocalciferol (Title) or alfacalcidol (Title) or alfacalcidol (Title) or paricalcitol (Title) or doxercalciferol (Title) or calcitriol (Title) or "25-Hydroxyvitamin D" (Title) \|  2#===== Diabete (Title) or "diabetes mellitus" (Title) or T2DM (Title) or hyperglycemia (Title) or hyperglycaemia (Title) or glucose (Title) or HbA1c (Title) or "glycated hemoglobin" (Title) or "insulin resistance" (Title) or "insulin sensitivity" (Title) or HOMA (Title) or "glucose homeostasis" (Title) or "insulin secretion" (Title) or insulin (Title) or "beta cell function" (Title) or "glycemic control" (Title) or "glucose tolerance" (Title) or "glucose metabolism" (Title) or "fasting blood glucose" (Title) or "Diabetes Mellitus" (Title) or Glycated Hemoglobin (Title)  3# AND #4 | 1761 |
| SCOPUS | ([TITLE-ABS-KEY] vitamin D supplementation" or [TITLE-ABS-KEY] "vitamin D" or [TITLE-ABS-KEY] "vitamin D2" or [TITLE-ABS-KEY] "vitamin D3" or [TITLE-ABS-KEY] cholecalciferol or [TITLE-ABS-KEY] ergocalciferol or [TITLE-ABS-KEY] alfacalcidol or [TITLE-ABS-KEY] alfacalcidol or [TITLE-ABS-KEY] paricalcitol or [TITLE-ABS-KEY] doxercalciferol or [TITLE-ABS-KEY] calcitriol or [TITLE-ABS-KEY] "25-Hydroxyvitamin D") AND ([TITLE-ABS-KEY] Diabete or [TITLE-ABS-KEY] "diabetes mellitus" or [TITLE-ABS-KEY] T2DM or [TITLE-ABS-KEY] hyperglycemia or [TITLE-ABS-KEY] hyperglycaemia or [TITLE-ABS-KEY] glucose or [TITLE-ABS-KEY] HbA1c or [TITLE-ABS-KEY] "glycated hemoglobin" or [TITLE-ABS-KEY] "insulin resistance" or [TITLE-ABS-KEY] "insulin sensitivity" or [TITLE-ABS-KEY] HOMA or [TITLE-ABS-KEY] "glucose homeostasis" or [TITLE-ABS-KEY] "insulin secretion" or [TITLE-ABS-KEY] insulin or [TITLE-ABS-KEY] "beta cell function" or [TITLE-ABS-KEY] "glycemic control" or [TITLE-ABS-KEY] "glucose tolerance" or [TITLE-ABS-KEY] "glucose metabolism" or [TITLE-ABS-KEY] "fasting blood glucose" or [TITLE-ABS-KEY] "Diabetes Mellitus" or [TITLE-ABS-KEY] Glycated Hemoglobin)) | 489 |

Supplementary file, Table 2: GRADE results

| **Summary of findings:** | | | | |
| --- | --- | --- | --- | --- |
|  | | | | |
| **Patient or population:** [patients with T2DM]  **Setting:** RCT studies  **Intervention:** Vitamin D supplementation  **Comparison:** Placebo | | | | |
| Outcomes | Relative effect (95% CI) | № of participants (studies) | Certainty of the evidence (GRADE) | Comments |
|  |  |  |  |  |
| The Effect of Vitamin D Supplementation on Glycemic Control and Cardiovascular Risk Factors in Type 2 Diabetes: An Updated Systematic Review and Meta-Analysis of Clinical Trials | **HbA1C** (SMD: -0.15; 95% CI: -0.29, -0.20) | 2190 interventions 2200 placebo  (45 interventional studies) | ⨁⨁⨁◯ moderate^a,b,c^ | The results also indicated that taking vitamin D supplements in the amount of 50,000 units had a significant effect on reducing the indicators related to diabetes control. |
|  | **FBS** (SMD: -0.28; %95 CI: -0.40, -0.15) | 1359 interventions 1399 placebo  (40 interventional studies) | ⨁⨁⨁◯ moderate^a,b,c^ |  |
|  | **SBP** (SMD: -0.06; %95 CI: -0.16, -0.05) | 1194 interventions 1269 placebo  (26 interventional studies) | ⨁⨁⨁◯ moderate^a,b,c^ |  |
|  | **LDL** (SMD: -0.11; %95 CI: -0.28, -0.05) | 802 interventions 843 placebo  (27 interventional studies) | ⨁⨁⨁◯ moderate^a,b,c^ |  |
|  | **HDL** (SMD: 0.13; 95% CI: 0.04, 0.29) | 1030 interventions 1069 placebo  (30 interventional studies) | ⨁⨁⨁⨁ high^a,b,c^ |  |
|  | **vitamin D levels** (SMD: 1.78; %95 CI: 1.53, 2.04) | 1601 intervention 1645 placebo  (48 interventional studies) | ⨁⨁⨁⨁ high^a,b,c^ |  |
| ***The risk in the intervention group** (and its 95% confidence interval) is based on the assumed risk in the comparison group and the **relative effect** of the intervention (and its 95% CI).  **CI:** confidence interval; **SMD:** Standardized Mean Difference  **FBS**: fasting blood glucose, **HbA1C**: Hemoglobin A1c, **LDL**: low-density lipoprotein, **HDL**: high-density lipoprotein, **SBP**: systolic blood pressure | | | | |
| **GRADE Working Group grades of evidence** **High certainty:** we are very confident that the true effect lies close to that of the estimate of the effect. **Moderate certainty:** we are moderately confident in the effect estimate: the true effect is likely to be close to the estimate of the effect, but there is a possibility that it is substantially different. **Low certainty:** our confidence in the effect estimate is limited: the true effect may be substantially different from the estimate of the effect. **Very low certainty:** we have very little confidence in the effect estimate: the true effect is likely to be substantially different from the estimate of effect. | | | | |
